# Comparison of Cancer Incidence between China and the USA

**DOI:** 10.3969/j.issn.2095-3941.2012.02.009

**Published:** 2012-06

**Authors:** Yong-chuan Wang, Li-juan Wei, Jun-tian Liu, Shi-xia Li, Qing-sheng Wang

**Affiliations:** Tianjin Medical University Cancer Institute and Hospital, Tianjin 300060, China

**Keywords:** neoplasms, epidemiology, China, USA

## Abstract

**Objective:**

The incidence of cancer varies around the globe, especially between less-developed and developed regions. The aim of this study is to explore differences in cancer incidence between China and the USA.

**Methods:**

Data were obtained from the GLOBOCAN 2008 database. Estimated numbers of new cancer cases in the USA were obtained from the American Cancer Society, while the numbers of cases in China, including those in urban and rural areas, were obtained from 36 cancer registries (2003-2005). Cancer incidence for major sites between China and the USA were analyzed.

**Results:**

In China, lung cancer was the predominant type of cancer detected in males; in females, breast cancer was the main type of cancer. Gastrointestinal cancers, such as those of the liver, stomach, and esophagus, were more commonly seen in China than in the USA. A significant difference in the incidence of melanoma of the skin was observed between China and the USA. During comparison of differences in the age-standardized rates by world population (ASRWs) of major cancer sites between the two countries, 4 sites in males (i.e., nasopharynx, esophagus, stomach, and liver) and 6 sites in females (i.e., nasopharynx, esophagus, stomach, liver, gallbladder, and cervix uteri) showed higher cancer incidence rates in China than in the USA.

**Conclusions:**

Significant differences in cancer incidence sites were found between the two countries. Cancer may be prevented through public education and awareness. Programs to promote cancer prevention in China, especially those of the lung, breast, and gastrointestinal region, must also be implemented.

## Introduction

Cancer incidence varies around the globe, especially between less-developed and developed regions. Demographic, ecological, environmental, cultural, and genetic variables all contribute to the heterogeneity of cancer incidence. Unfortunately, little information is available about cancer in the majority of less-developed countries ^[^^1^^]^. China, one of the biggest less-developed countries, has an immense cancer problem, and a cancer control system must be established and refined at the state level to suit China’s current socioeconomic status. Thus, strategies for cancer prevention and control implemented in the USA are valuable models for China. Patterns in cancer incidence can provide important insights into the impact of lifestyle on cancer development, and a comparison of cancer incidence between China and the USA can provide useful information which will help determine and meet requirements for cancer prevention and control.

## Materials and Methods

The data used in this study were based on GLOBOCAN 2008. The methods used to estimate specific cancer problems in a country are similar to those used in the GLOBOCAN 2002 study ^[^^1^^]^, and have been described in detail elsewhere^[^^2^^]^. The most recent disease rates available were summarized according to the population of the country in 2008. Estimated national mortalities from various cancers in China were converted into degrees of incidence using modeling. Datasets of age, sex, and site-specific incidence mortality ratios derived from recorded data in 36 cancer registries (2003-2005) were utilized in the model ^[^^3^^]^. The population covered by these registration areas totaled 128,213,000 (including 55,694,000 people in urban areas and 72,519,000 people in rural areas). Estimated numbers of new cancer cases for all ages in the USA were obtained from the American Cancer Society.

Numbers of incidence and age-standardized rates by world population (ASRWs) were analyzed. ASRW ratios were calculated as:

ASRW_China_< ASRW_USA_: ASRW Ratio=-1/(ASRW_China_ /ASRW_USA_)

ASRW_China_> ASRW_USA_: ASRW Ratio=ASRW_China_ /ASRW_USA_

## Results

**Tables 1**** and ****2** show the number, proportion, and ASRWs of major types of cancer in China and the USA. In China, lung cancer ranked the first among all sites, followed by cancers of the stomach, liver, colorectum, and breast. Lung cancer (ASRW=45.9 per 100,000) was the leading cancer in males, followed by cancers of the stomach (41.3), liver (37.4), esophagus (22.9), and colorectum (16.3). In females, breast cancer (21.6) ranked the first, followed by cancers of the lung (21.3), stomach (18.5), liver (13.7), and colorectum (12.2).

**Table 1:** **.** Estimated cancer incidence (2008): China and the USA, male, all ages (ASRW and proportion per 100,000).

Cancer	China				USA				ASRW ratio (%)
	Number	%	ASRW		Number	%	ASRW		
Lip, oral cavity	10427	0.6	1.4		15817	2.1	7.3		-5.2
Nasopharynx	22317	1.4	2.8		1352	0.2	0.7		4.0
Other pharynx	4515	0.3	0.6		8144	1.1	3.9		-6.5
Esophagus	175863	10.8	22.9		12970	1.7	5.8		4.0
Stomach	315843	19.5	41.3		13189	1.8	5.7		7.3
rectum	125461	7.7	16.3		79271	10.6	34.1		-2.1
Liver	292966	18.1	37.4		15192	2.0	7.0		5.3
Gallbladder	11434	0.7	1.5		4501	A0.6	1.9		-1.3
Pancreas	24841	1.5	3.2		18771	2.5	8.0		-2.5
Larynx	17077	1.1	2.3		9681	1.3	4.4		-1.9
Lung	351713	21.7	45.9		114691	15.4	49.5		-1.1
Melanoma of skin	2281	0.1	0.3		34949	4.7	16.3		-54.3
Prostate	33802	2.1	4.3		186320	25.0	83.8		-19.5
Testis	2795	0.2	0.4		8090	1.1	5.1		-12.8
Kidney	21269	1.3	2.8		34638	4.6	16.1		-5.8
Bladder	42686	2.6	5.5		51231	6.9	21.1		-3.8
Brain, nervous system	33244	2.0	4.3		11779	1.6	6.3		-1.5
Thyroid	6286	0.4	0.8		8931	1.2	4.6		-5.8
Hodgkin lymphoma	3740	0.2	0.5		4400	0.6	2.6		-5.2
Non-Hodgkin lymphoma	19205	1.2	2.5		35453	4.8	16.3		-6.5
Multiple myeloma	3274	0.2	0.4		11188	1.5	4.8		-12.0
Leukemia	38635	2.4	5.3		25179	3.4	12.1		-2.3

**Table 2:** **.** Estimated cancer incidence (2008): China and the USA, female, all ages (ASRW and proportion per 100,000).

Cancer	China				USA				ASRW ratio (%)
	Number	%	ASRW		Number	%	ASRW		
Lip, oral cavity	5483	0.5	0.7		7355	1.1	2.8		-4.0
Nasopharynx	10784	0.9	1.4		565	0.1	0.3		4.7
Other pharynx	1709	0.1	0.2		2077	0.3	0.8		-4.0
Esophagus	83372	7.0	10.5		3500	0.5	1.2		8.8
Stomach	148596	12.4	18.5		8310	1.2	2.8		6.6
Colorectum	95852	8.0	12.2		74610	10.8	25.0		-2.0
Liver	109242	9.1	13.7		6182	0.9	2.2		6.2
Gallbladder	14418	1.2	1.8		5019	0.7	1.6		1.1
Pancreas	19376	1.6	2.4		18914	2.7	6.1		-2.5
Larynx	4058	0.3	0.5		2570	0.4	1.1		-2.2
Lung	170337	14.3	21.3		100330	14.5	36.2		-1.7
Melanoma of skin	1544	0.1	0.2		27531	4.0	12.8		-64.0
Breast	169452	14.2	21.6		182460	26.4	76.0		-3.5
Cervix uteri	75434	6.3	9.6		11069	1.6	5.7		1.7
Corpus uteri	86066	7.2	11.1		40102	5.8	16.5		-1.5
Ovary	28739	2.4	3.8		21652	3.1	8.8		-2.3
Kidney	11239	0.9	1.5		22040	3.2	8.7		-5.8
Bladder	12241	1.0	1.5		17581	2.5	5.8		-3.9
Brain, nervous system	33210	2.8	4.5		10029	1.4	4.9		-1.1
Thyroid	15597	1.3	2.1		28411	4.1	15.1		-7.2
Hodgkin lymphoma	1860	0.2	0.3		3820	0.6	2.2		-7.3
Non-Hodgkin lymphoma	12811	1.1	1.7		30673	4.4	11.5		-6.8
Multiple myeloma	2635	0.2	0.3		8732	1.3	3.0		-10.0
Leukemia	32191	2.7	4.7		19090	2.8	7.9		-1.7

In ranking cancer cases in China, the most common types of cancer in males were those of the lung (351,713 cases, 21.7% of all cancers), stomach (315,843 cases, 19.5%), and liver (292,966 cases, 18.1%). In the USA, the most common cancers in males included those of the prostate (186,320 cases, 25.0%), lung (114,691 cases, 15.4%), and colorectum (79,271 cases, 10.6%). In China, the most common female cancers included those of the lung (170,337 cases, 14.3%), breast (169,452 cases, 14.2%), and stomach (148,596 cases, 12.4%); in the USA, the most common types of cancer in females included those of the breast (182,460 cases, 26.4%), lung (100,330 cases, 14.5%), and colorectum (74,610 cases, 10.8%).

The incidence rate of melanoma was significantly different between China and the USA. The ASRW of melanoma in the USA for males was 54.3 times greater than that in China; for females, the ASRW was about 64 times greater in the USA. In addition, the incidence rates of multiple myeloma (ASRW=12.0 for males and 10.0 for females), prostate cancer (ASRW=19.5), and testicular cancer (ASRW=12.8) were significantly different between the two countries.

**Figure 1:**
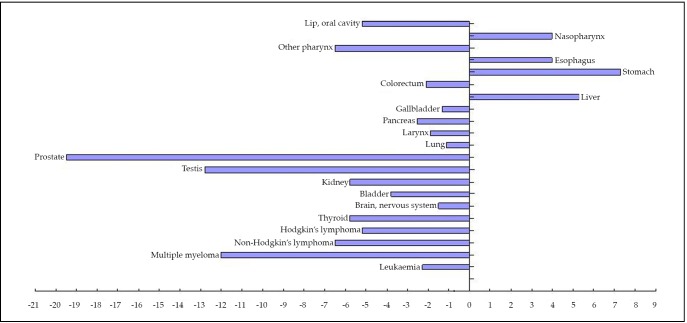
Estimated cancer incidence (2008): China and the USA, male, all ages.

**Figure 2:**
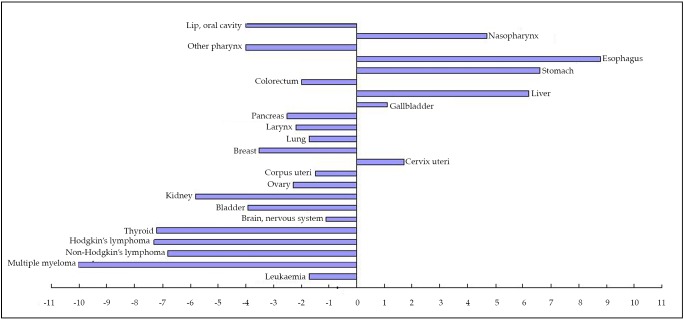
Estimated cancer incidence (2008): China and the USA, female, all ages.

In comparing differences in the ASRWs of major cancer sites between the two countries, 4 sites in males (i.e., nasopharynx, esophagus, stomach, and liver) and 6 sites in females (i.e., nasopharynx, esophagus, stomach, liver, gallbladder, and cervix uteri) showed higher incidence rates in China than in the USA, as shown in **Figures 1**** and ****2**.

## Discussion

Cancer is currently a global issue. Millions of people die of cancer every year. Information on the cancer problem and epidemic trends in different areas and populations is very important. Extensive analysis in this field serves as the foreground for creating a national strategy for cancer prevention and control.

The GLOBOCAN Project is a network established by the International Agency for Research on Cancer of the World Health Organization that aims to provide contemporary estimates of the incidence of mortality and prevalence of major types of cancer at a national level in 184 countries all over the world. The most updated cancer data obtained from GLOBOCAN for these areas studied were estimates for 2008. For GLOBOCAN 2008, the incidence data were derived from population-based cancer registries, which may cover entire national populations but more often involve smaller and sub-national areas. In China, only major cities are usually included. While the quality of information in China may not be sufficient, the information has unique relevance as it remains the only unbiased source of data available on the profiles of various cancers.

Lung cancer is a major health issue both in China and in the USA. An earlier study reported a 1.63% increase in lung cancer incidence per year from 1988 to 2005 in China ^[^^4^^]^. The mortality rate of lung cancer was 30.84 per 100,000 in 2005, rising by 465% over the past 30 years ^[^^5^^]^. In the USA, an analysis of long-term trends (1975-2005) showed that both the incidence and death rate of lung cancer have decreased statistically in men and women. This decrease is believed to be driven largely by tobacco control in the country ^[^^6^^]^. Data show that lung cancer is an ongoing health issue in China and that the problem is becoming more serious very year. Effective long-term strategies on lung cancer prevention and control are important, especially since the strengthening and maintenance of tobacco control programs in China is faced with continuing obstacles. Yano et al. ^[^^7^^]^ designed a case-control study of 1,139 asbestos workers with 41 males identified with lung cancer in China in 2001. Findings confirmed the strong association between exposure to chrysotile asbestos and lung cancer risk and supported the important interactive effect of asbestos exposure and smoking.

Breast cancer remains the most common cancer for females in both China and the USA, with ASRWs of 21.6 and 76.0 per 100,000, respectively. In a comparative study between Tianjin and New York, the cancer pattern in Chinese immigrants was found to differ in the immigrated city ^[^^8^^]^, indicating that cancer risks could vary when the environment changes. Unprecedented declines were observed in invasive breast cancer rates in the USA between 2001 and 2004, with Hausauer et al. ^[^^9^^]^ reporting that incidence fell by 13.2% and that greater reductions were observed in women living in urban areas (-13.8%) than in rural areas (-7.5%). These patterns reflect the major influence of hormone therapy use and screening patterns in cancer reduction. Other findings from previous studies support the need for breast cancer screening programs (including regular clinical breast examinations and mammography), as well as public education and awareness regarding early detection of breast cancer ^[^^10^^]^. In one such study, breast cancer survival rates in two populations, between Ardabil, Iran and British Columbia, Canada, were compared, and overall findings indicated that breast cancer patients in British Columbia had a survival of one year higher than patients in Ardabil for each age group under 60 years old. All of these findings indicate that more efforts should be exerted toward cancer-preventive measures, including cancer screening performed by the census to ensure early detection of cancer, population health education, anti-cancer knowledge popularization, and identification and correction of unhealthy lifestyles and habits that may result in cancer.

Clear differences in cancer incidence rates between China and USA were observed, particularly for melanoma of the skin, which is usually associated with race and sunburn. For example, the ASRW of prostate cancer in the USA was found to be about 83.8 per 100,000, while that in China was only 4.3. Similar but less attenuated patterns were seen for multiple myeloma and male testicular cancers. The incidence of gastrointestinal cancers, including carcinomas of the stomach, liver, and esophagus was higher in China than in the USA. Wiggins ^[^^11^^]^ analyzed cancer incidence rates in the USA and found that incidence rates varied among American Indian and Alaska Native populations and often differed from rates among non-Hispanic whites. This finding highlighted opportunities for cancer control and prevention but not the misclassification of races. Wiggins discussed the possible effects of environmental factors, lifestyle, and eating habits, among other cancer factors. Micheli et al. ^[^^12^^]^ reported large differences in the prevalence of some cancer in countries included in the European registry. Richer areas of Europe had higher prevalence rates, suggesting that incidence varied with economic development.

In China, several types of cancers were found to be more common, including liver and esophageal cancers, which was related largely to conditions of relatively less development. The high incidence of some cancers, including those of the lung, colorectum, and female breast, is associated with economically developed societies. Cancer incidence has significantly increased since the 1970s, and cancer mortality has become the second leading cause of death in the Chinese population and the leading cause of death in urban areas^[^^13^^]^. Cancer has seriously affected the national economy, social development, and national public health system. This information is useful in making plans for health promotion, cancer prevention, and control strategies and serves as a guide for future scientific research.

Obtaining complete, accurate, and controlled information on cancer incidence and relevant factors in cancer prevention and control is highly significant. While this study utilized updated information and data, it also presented several limitations, such as non-inclusion of detailed information regarding coverage area and age data in cancer registries and coverage of only smaller and sub-national areas instead of the entire national population of China. Mortality rates and other common statistical indicators must also be analyzed in future studies.

## Conclusion

The most commonly found cancers vary between China and the USA. Cancers of the lung, breast, and gastrointestinal region, including those of the stomach, liver, esophagus, and colorectum, are more commonly seen in China than in the USA. Thus, plans should be carefully made to develop health plans and cancer prevention and screening strategies to guide scientific research applicable to China’s socioeconomic status and current situation. Public education, including antismoking and tobacco control, and awareness campaigns regarding early detection of cancer are also very important to minimize cancer incidence.
